# Classification of Functional Metagenomes Recovered from Different Environmental Samples

**DOI:** 10.6026/97320630015026

**Published:** 2019-01-31

**Authors:** Zobaer Akond, Mohammad Nazmol Hasan, Md.Jahangir Alam, Munirul Alam, Md.Nurul Haque Mollah

**Affiliations:** 1 Bioinformatics Lab, Department of Statistics, University of Rajshahi, Rajshahi-6205,Bangladesh; 2 Institute of Environmental Science,University of Rajshahi-6205,Bangladesh; 3 Agricultural Statistics and Information and Communication Technology (ASICT) Division,Bangladesh Agricultural Research Institute(BARI),Joydebpur,Gazipur-1701,Bangladesh; 4 Emerging Infections, Infectious Diseases Division,International Centre for Diarrheal Disease Research,Bangladesh (icddr,b); 5 Bangabandhu Sheikh Mujibur Rahaman Agricultural University,Joydebpur,Gazipur-1706, Bangladesh

**Keywords:** metagenomes, classification, true positive rate, false positive rate, misclassification error, beta-t random forest

## Abstract

Classification of functional metagenomes from the microbial community plays the vital role in the metagenomics research. In this paper, an investigation was made to study the performance of beta-t random forest classifier for classification of metagenomics data. Nine key functional meta-genomic variables were selected using the beta-t test statistic from the 10 different microbial community using p-value at 5% level of significance. Then beta-t random forest classifier showed the higher accuracy (96%), true positive rate (96%) and lower false positive rate (5%), false discovery rate (5%) and misclassification error rate (5%) for classification of metagenomes. This method showed the better performance compare to Bayes, SVM, KNN, AdaBoost and LogitBoost).

## Background

Metagenomics is one of the important research wings in bioinformatics for studying the microbial community available in different environments. The classification of functional metagenomes is the big statistical challenge from the different sources of metagenome dataset. The classification of potential metabolic function from microbial community using metagenomic information is an important task of metagenomics research. The different microbial process has different metagenomic function for several environments [1]. Metagenomics is the complete scheme of microbial activity and gives the easier interpretation of thousands of proteins using BLAST matches algorithm [2]. There are many web tools available for statistical analysis of metagenomic dataset but not all the analysis tools provide accurate and valid results [3].

Some traditional multivariate statistical methods such as principal component analysis (PCA), multidimensional scaling (MDS), and canonical discriminate analysis (CDA) are often used for analysis of genomic data and microbial community [4]. The multivariate statistical techniques are plays vital role for classification and visualization of metagenomics data from several microbial community. The metagenomic data profiling from the different environments and its classification is important for separation of functional metagenomes. The MetaGUN is the three-stage gene selection method for gene prediction for metagenomic fragments using support vector machine (SVM) [5, 7]. To explore the universe of metagenome, k-nearest neighbor method is significant for the several microbial communities [6]. AdaBoost is the efficient method for analyzing the gigantic metagenomic data and it is challenging task for bioinformaticians/computer scientists [8]. The prediction of ribosomal protein in plants, the machine learning method Random Forest is very much useful [9-12]. The statistical test is very important for the identification of potential metabolic function within and between environments based on the different microbial community. Random forest method is efficient for the robust classification of high dimensional complexity data like as the microbial community data. It is the ensemble learning method for classification and regression multiple patterns datasets. High dimensional dataset with large number of features or metabolic functions or metabolic variables is a very basic problem. Therefore, it is essential to select the proper feature selection method for classification of large dimensional metagenomics dataset. In this study, we used beta t-statistic for feature selection of metagenomic data from the several microbial community then applied random forest algorithm for efficient classification of functional metagenomes.

## Methodology

### Dataset:

The dataset in this study were collected from the previously published article [17]. The dataset contains 212 microbial metagenomes generated from the 10 different environments with 26 metabolic functions.

### Model:

**Figure 1:**
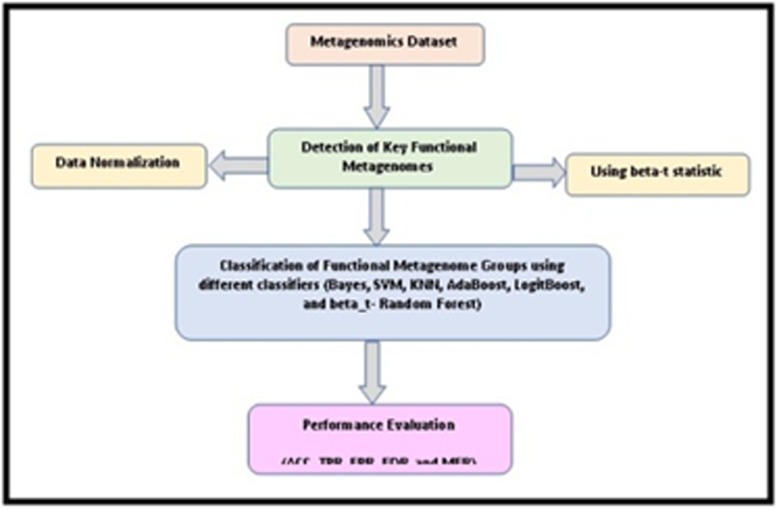
Schematic diagram of this study

The Bayesian classifier is generally known as a simple probabilistic classifier. The sequence features were used for the input X = (x1x2...xp) to the Bayesian classifier. For each metagenome, our Bayesian classifier produced a multiclass and the Bayesian classifier was trained using a set of labeled training dataset (X, C). The Support Vector Machine (SVM), K-nearest Neighbor (KNN), AdaBoost, LogitBoost [13] Random Forest [15] Beta-t statistic [16] are used for classification and comparison of functional metagenomes from the different microbial community (Figure 1). All the computational analysis conducted in this study using R-statistical programming language (https://www.r-project.org/) [14].

## Results and Discussion:

To identify the key functional metagenomes, we used the beta t-test statistic. This method is described in details in the previously published paper [16]. Using the method we select the top nine key functional metagenomes (AAD, CDCC, CVPGP, DNAM, MT, MC, NN, Plasmids and SM) based the on the p-values at 5% level of significance (Table 1). The key functional metagenomes are abbreviated in the alphabetical letter case those are selected from the 10 different microbial community and 212 metagenomes.

**Figure 2:**
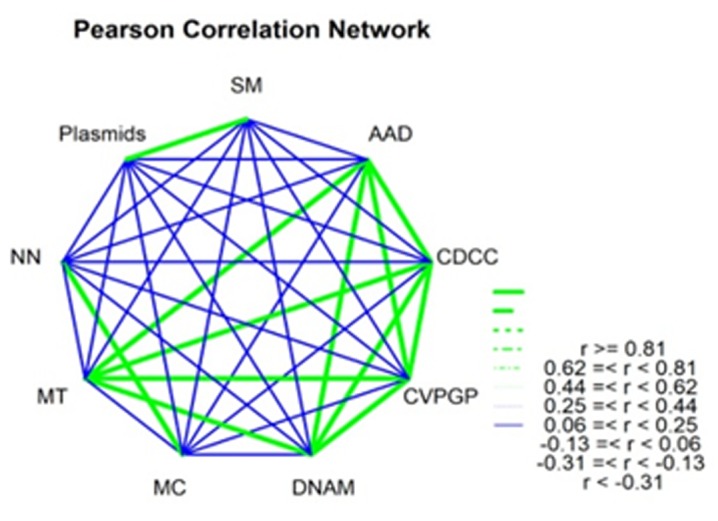
Pearson correlation network for nine key functional metagenomes

The Pearson correlation network plot (Figure 2) showed the correlation among the key functional metagenom. The ADD (amino acids and derivatives) is strongly correlated (>0.81) with the other metagenomes CDCC (Cell Division and Cell Cycle), CVPGP (Cofactors Vitamins Prosthetic Groups Pigments), DNAM (DNA Metabolism) and MT (Membrane Transport). The highly positive correlations among the meta-genomic variables imply that they are similar in direction from AAD functional metagenomes. On the other hand, ADD is negatively correlated with the MC (Motility and Chemotaxis), NN (Nucleosides and Nucleotides), Plasmids, and SM (Sulfur Metabolism). The opposite relationship existed among the functional metagenomes in the different microbial community.

To investigate the performance of the different classifiers we divided full dataset into three different parts using the cross validation (CV) method such as 10-fold, 5-fold, and 3-fold cross validation dataset and checking the performance. In case of full dataset, performance of different classifiers (Bayes, SVM, KNN, AdaBoost, LogitBoost and Beta-t Random Forest) is shown in the Table 2. The performance measure of all the methods using accuracy (AAC), true positive rate (TPR), false positive rate (FPR), false discovery rate (FDR), and misclassification error rate (MER). Bayes classifier showed the lowest performance in terms of ACC (57%), TPR (57%), FPR (42%), FDR (48%), and MER (43%) whereas the highest performance is observed for beta-t Random Forest in terms of ACC, TPR, FPR, FDR and MER with the results of 94%, 94%, 5%, 6% and 6% respectively. Finally, we showed that beta-t Random Forest provided the better performance for full dataset. For the 10-fold cross validation dataset, the Bayes classifier showed the lowest performance and LogitBoost and beta-t Random Forest showed approximately equal performance but eventually beta-t Random Forest was considered as better classifier than the other methods. In case of 5-fold and 3-fold cross validation dataset, it is found that the beta-t Random Forest method showed better ACC, TPR, FPR, FDR, and MER respectively.

**Figure 3:**
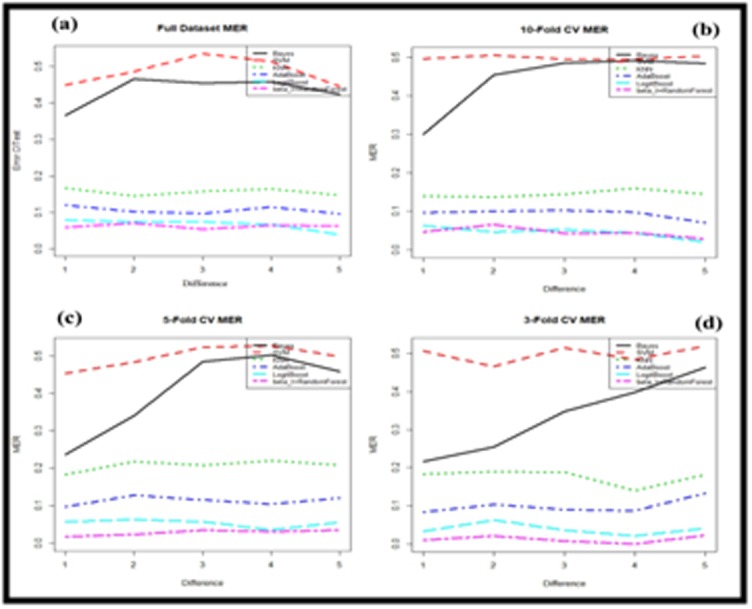
Misclassification error rate (a) Full dataset, (b) 10-fold cross validation, (c) 5-fold cross validation and (d) 3-fold cross validation for different classifiers.

From the Figure 3 it is revealed that among the misclassification error rate (MER) of the six different classifiers, the SVM classifier provided the highest MER and beta-t Random Forest showed the lowest MER for full dataset. Similarly, for other datasets (10-fold, 5- fold, and 3-fold CV) SVM also showed the highest MER and beta-t Random Forest provided the lowest MER. It is however demonstrated that the beta-t Random Forest showed the lowest MER for all datasets.

**Figure 4:**
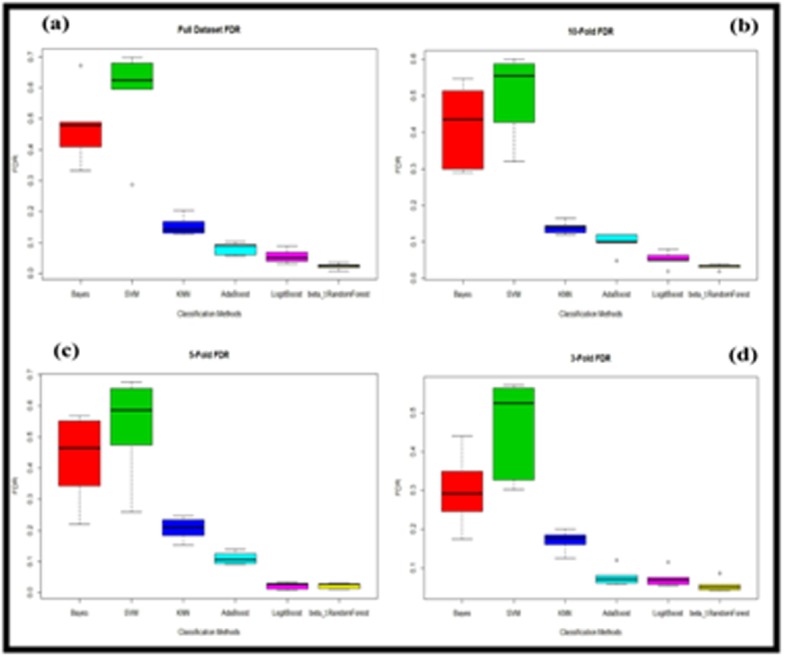
False discovery rate (a) Full dataset, (b) 10-fold (c) 5-fold and (d) 3-fold cross validation for different classification methods of metagenome dataset.

The false discovery rate (FDR) was calculated for each of the dataset. Figure 4 illustrates that SVM produced largest FDR for all datasets followed by Bayes classifier and KNN. On the other hand, among these six classifiers, the beta-t Random Forest produced lowest FDR to classify the functional metagenomes from several microbial communities.

**Figure 5:**
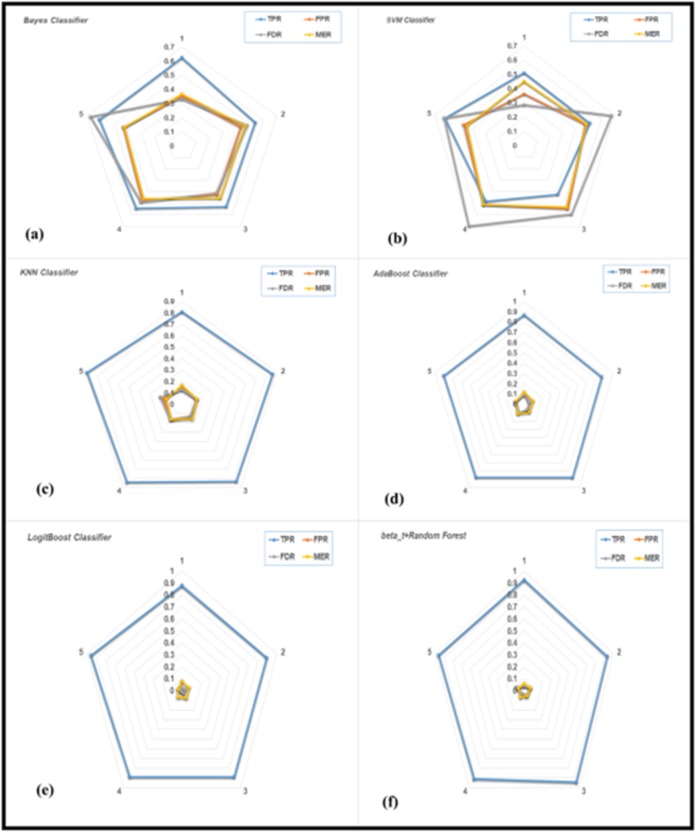
The average classification performances of (a) Bayes, (b) SVM, (c) KNN, (d) AdaBoost, (e) LogitBoost and (f) beta-t+Random Forest classification methods for metagenome dataset.

The radar plot (Figure 5) shows the different performance measurement methods for popular classifiers in the literature to classify the functional metagenomes from the different microbial community. The beta-t Random Forest classifier showed the highest TPR and lowest FDR and MER for classification of the metagenomes.

## Conclusion

Classification of the metagenomic data obtained from different microbial community is an important task in the context of their associated functional metagenomic variables. In this study the betat random forest classifier showed the lowest FDR and MER along with highest TPR in all cases of data compared to Bayes, SVM, KNN, AdaBoost and LogitBoost classifiers. Therefore, the beta-t random forest classifier is considered the best classifier in grouping the metagenomes derived from different environmental samples.

## Figures and Tables

**Table 1:** Key Metabolite functions selected by the beta-t test statistic

Key Metabolite Functions	Metabolite Function Abbreviation	p-value*
Amino Acids and Derivatives	AAD	0.041
Cell Division and Cell Cycle	CDCC	0.034
Cofactors Vitamins Prosthetic Groups Pigments	CVPGP	0.043
DNA Metabolism	DNAM	0.005
Membrane Transport	MT	0.015
Motility and Chemotaxis	MC	0.025
Nucleosides and Nucleotides	NN	0.007
Plasmids	Plasmids	0.014
Sulfur Metabolism	SM	0.026

**Table 2:** Classification performance of different classifiers

Methods		ACC	TPR	FPR	FDR	MER
	Full Dataset					
Bayes		0.566	0.569	0.422	0.477	0.434
SVM		0.514	0.509	0.475	0.577	0.486
KNN		0.844	0.849	0.149	0.154	0.156
AdaBoost		0.894	0.878	0.083	0.081	0.106
LogitBoost		0.933	0.901	0.025	0.024	0.067
Beta_t+Random Forest		0.937	0.935	0.054	0.056	0.063
	10-Fold Cross Validation					
Bayes		0.557	0.549	0.421	0.417	0.443
SVM		0.501	0.506	0.509	0.498	0.499
KNN		0.855	0.852	0.138	0.139	0.145
AdaBoost		0.907	0.912	0.094	0.097	0.093
LogitBoost		0.955	0.944	0.032	0.032	0.045
Beta_t+Random Forest		0.955	0.962	0.05	0.052	0.045
	5-Fold Cross Validation					
Bayes		0.596	0.592	0.402	0.429	0.404
SVM		0.503	0.503	0.485	0.53	0.497
KNN		0.793	0.798	0.202	0.205	0.207
AdaBoost		0.887	0.892	0.107	0.111	0.113
LogitBoost		0.946	0.922	0.023	0.022	0.054
Beta_t+Random Forest		0.972	0.968	0.021	0.022	0.028
	3-Fold Cross Validation					
Bayes		0.664	0.664	0.329	0.3	0.336
SVM		0.502	0.496	0.486	0.458	0.498
KNN		0.824	0.823	0.168	0.169	0.176
AdaBoost		0.901	0.886	0.078	0.078	0.099
LogitBoost		0.962	0.939	0.011	0.011	0.038
Beta_t+Random Forest		0.988	0.98	0.003	0.003	0.012
